# Reinvestigation of Absorption Spectroscopic Thermal Dynamics of Archaerhodopsin 3 Based Voltage Sensor QuasAr1

**DOI:** 10.3390/bioengineering12121293

**Published:** 2025-11-24

**Authors:** Alfons Penzkofer, Arita Silapetere, Peter Hegemann

**Affiliations:** 1 Fakultät für Physik, Universität Regensburg, Universitätsstraße 31, D-93053 Regensburg, Germany; 2 Experimentelle Biophysik, Institut für Biologie, Humboldt Universität zu Berlin, Invalidenstraße 42, D-10115 Berlin, Germany

**Keywords:** QuasAr1, Archaerhodopsin, fluorescent voltage sensor, absorption spectroscopic characterization, protein aggregation, Rayleigh and Mie scattering, numerical simulation of thermal dynamics, ground-state isomerization, ground-state deprotonation

## Abstract

The long-time absorption spectroscopic development of the genetically encoded microbial rhodopsin fluorescent voltage indicator QuasAr1 at room temperature in the dark was reinvestigated, mainly theoretically. The data analysis indicates protein aggregation within one day to some ten-nanometer sized Mie scattering particles. The absorption coefficient spectra can be deduced from measured attenuation coefficient spectra by scattering contribution subtraction. The initially present protonated retinal Schiff base (PRSB) Ret_580 isomerized and then deprotonated to neutral retinal Schiff base (RSB). One part of Ret_580, Ret_580_I_, (fraction 43%), isomerized moderately fast to Ret_500 which then deprotonated to neutral retinal Schiff base Ret_405 (time constant ≈ 1000 h). The other part of Ret_580, Ret_580_II_, (fraction 57%), isomerized slowly to Ret_460 which deprotonated to Ret_340 (time constant ≈ 400 h). The dynamics are described by a differential equation system which is solved numerically. Reaction parameters are determined by fitting the simulations to the experimental results.

## 1. Introduction

The protein QuasAr1 was derived from Archaerhodopsin 3 (Arch) of *Halorubrum sodomense* [1,2,3] by directed evolution [4] (QuasAr1 = Arch P60S-T80S-D106H-F161V). QuasAr1 is one member of a wide group of microbial rhodopsin-based fluorescent genetically encoded voltage indicators (GEVIs) [5,6,7,8,9,10,11,12] applied in optical electrophysiology of biological membrane potential determination [13,14]. QuasAr1 was used and characterized as a transmembrane voltage sensor in neuroscience [4,15] and cardiac science [16]. The thermal absorption and emission spectroscopic behavior of QuasAr1 was investigated in [17]. Here the long-time absorption development at room temperature in the dark was analyzed in detail and the model of thermal dynamics presented in [17] was modified. The temporal Rayleigh and Mie scattering evolution was studied. The absorption coefficient spectra development was extracted from measured attenuation coefficient spectra by subtracting scattering contributions. The absorption spectra development was determined by isomerization of the originally present protonated retinal Schiff base (PRSB) and deprotonation of the formed isomers to neutral retinal Schiff base (RSB). The isomerization and deprotonation dynamics were analyzed by a differential equation system which was solved numerically. Reaction parameters were extracted by numerical fitting to experimental results.

**Figure 1:**
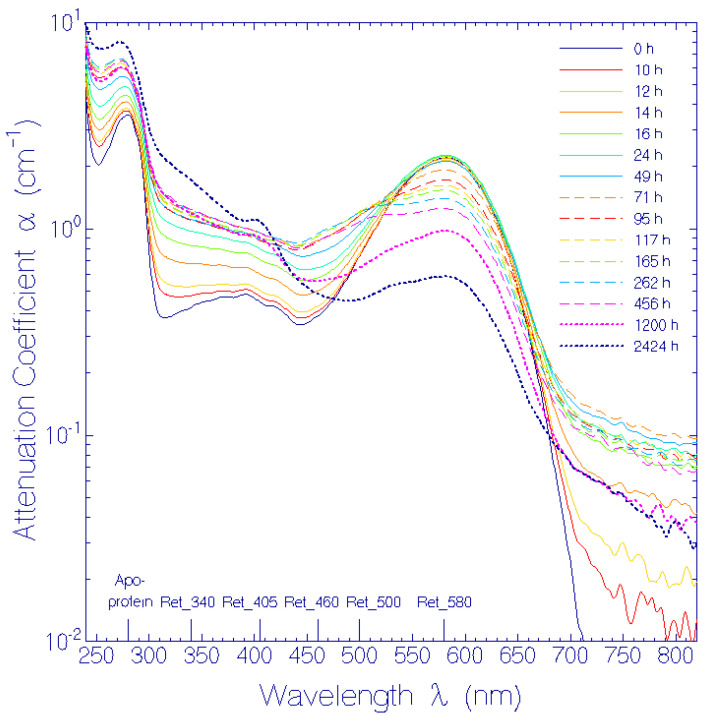
Temporal development of attenuation coefficient spectra α(λ) of QuasAr1 in pH 8 Tris buffer at room temperature (ϑ = 21–25 °C) in the dark. The storage times are listed in the legend.

**Figure 2:**
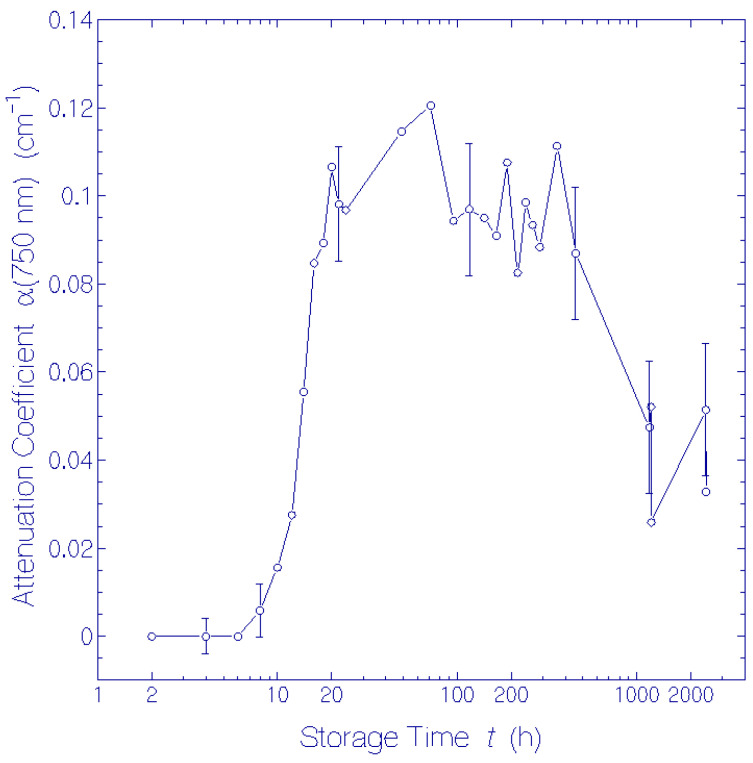
Attenuation coefficient α(750 nm) development versus storage time *t* at room temperature in the dark of QuasAr1 in pH 8 Tris buffer.

**Figure 3:**
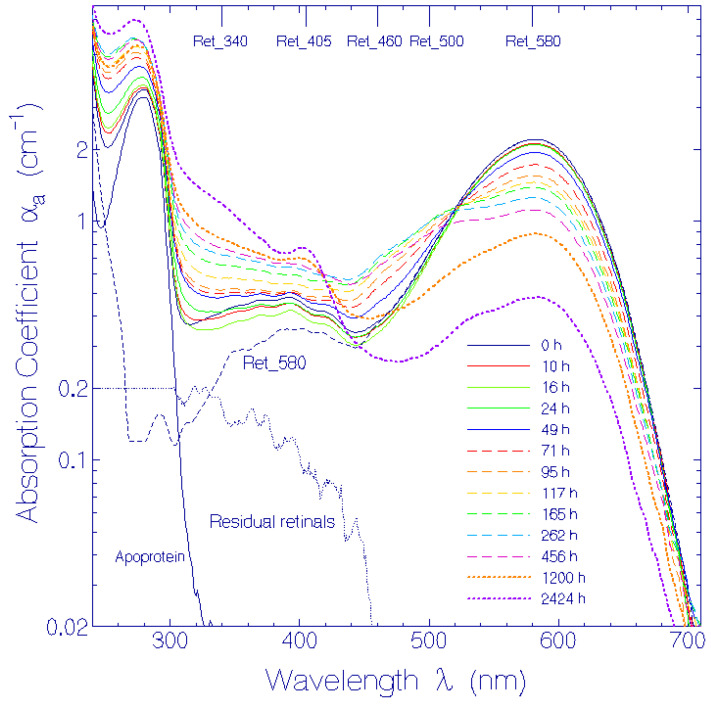
Determined absorption coefficient spectra α_a_ of QuasAr1 in pH 8 Tris buffer at room temperature in the dark. The storage time *t* is listed in the legend. The absorption coefficient spectra $\alpha_{a,\mathrm{residual}\;\mathrm{retinals}}(\lambda ,t=0)$, $\alpha_{a,\mathrm{apoprotein}}(\lambda ,t=0)$, and $\alpha_{a,\mathrm{Ret}\_580}(\lambda ,t=0)=\alpha_{a}(\lambda ,t=0)-\alpha_{a,\mathrm{residual}\;\mathrm{retinals}}(\lambda ,t=0)-\alpha_{a,\mathrm{apoprotein}}(\lambda ,t=0)$ are included.

**Figure 4:**
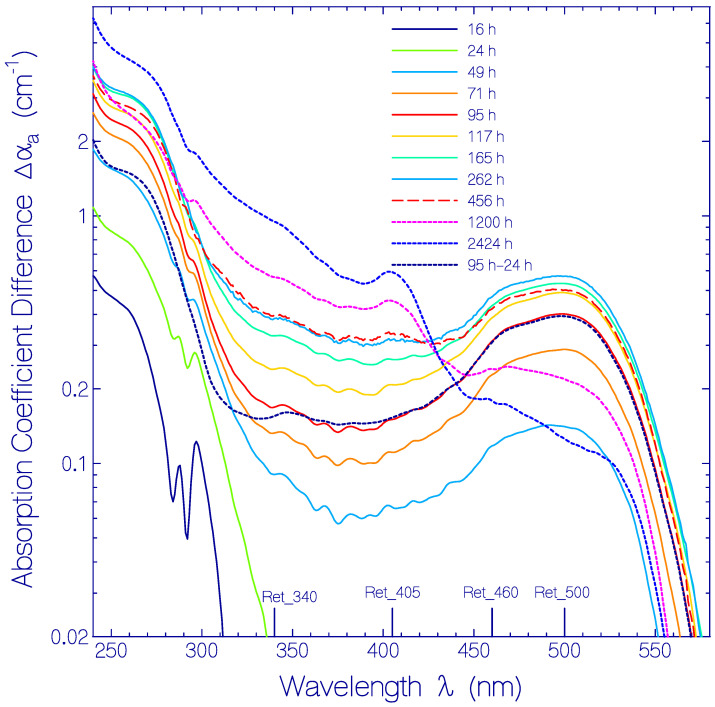
Temporal development of absorption of decomposition products of Ret_580: PRSB isomers Ret_500, Ret_460, and deprotonated RSB components Ret_405 and Ret_340. The absorption coefficient difference $\Delta \alpha_{a}(\lambda ,t)=\alpha_{a}(\lambda ,t)-\alpha_{a,\mathrm{Ret}\_580}(\lambda ,t)-\alpha_{a,\mathrm{residual}\;\mathrm{retinals}}(\lambda ,t=0)-\alpha_{a,\mathrm{apoprotein}}(\lambda ,t=0)$ is displayed.

**Figure 5:**
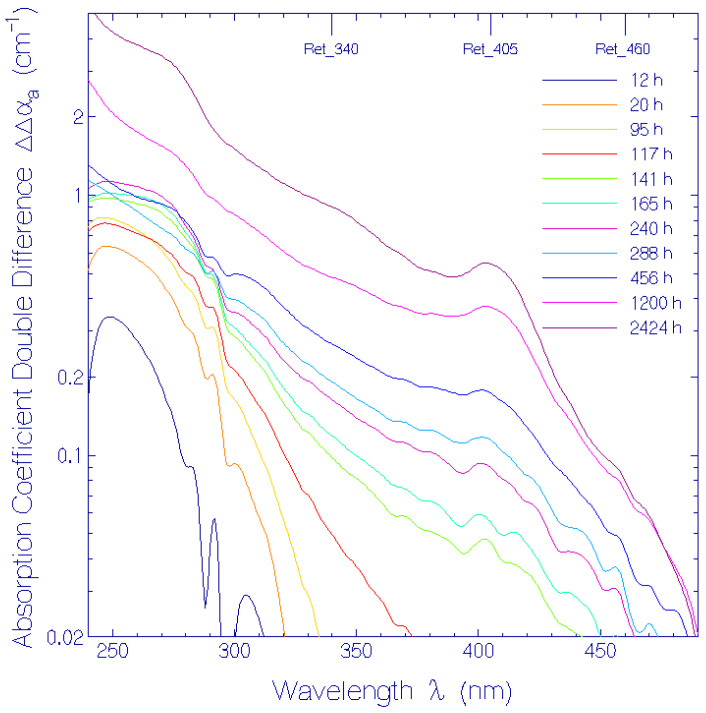
Temporal development of absorption of isomer product Ret_460, and deprotonation products Ret_405 and Ret_340. The absorption coefficient double difference $\Delta \Delta \alpha_{a}(\lambda ,t)=\Delta \alpha_{a}(\lambda ,t)-\alpha_{a,\mathrm{Ret}\_500}(\lambda ,t)$ is displayed.

The experimental attenuation coefficient spectra development, α(λ, *t*), at room temperature presented in ref. [17] (Figure S6) was redrawn (present Figure 1). The long-wavelength attenuation development (Figure 2), due to thermally driven protein aggregation, was analyzed, applying the Mie scattering theory. The absorption coefficient spectra development, α_a_(λ, *t*), was deduced from the attenuation coefficient spectra development by empirical subtraction of the Mie scattering contribution, α_s_(λ, *t*) (Figure 3). The initial absorption coefficient spectrum, originating from contributions of (i) protonated retinal Schiff base (PRSB) Ret_580 (absorption maximum at wavelength λ = 580 ± 2 nm), (ii) residual retinals (weak absorption contribution below 450 nm) and (iii) apoprotein (peak absorption around 280 nm), evolved by Ret_580 degradation (ground-state thermal isomerization). The speed of Ret_580 isomerization changed with time (Figure S11): The fast component, Ret_580_I_, built up a new absorption band peaking at λ = 500 ± 5 nm (Ret_500), and the slow component, Ret_580_II_, formed a weak absorption band at λ = 460 ± 10 nm (Ret_460). The Ret_580 degradation spectra development is shown in Figure 4 by presenting the absorption coefficient difference spectra $\Delta \alpha_{a}(\lambda ,t)=\alpha_{a}(\lambda ,t)-\alpha_{a,\mathrm{Ret}\_580}(\lambda ,t)-\alpha_{a,\mathrm{residual}\;\mathrm{retinals}}(\lambda ,0)\;-\alpha_{a,\mathrm{apoprotein}}(\lambda ,0)$. The initial $\Delta \alpha_{a}(\lambda ,t)$ development is determined by Ret_500 formation. At longer times, the $\Delta \alpha_{a}(\lambda ,t)$ development is determined by Ret_500 degradation (deprotonation to Ret_405 with absorption band peaking at λ = 405 ± 5 nm) as well as Ret_460 formation and degradation (deprotonation to Ret_340 with the absorption band peaking at λ = 340 ± 15 nm). The absorption coefficient double difference spectra $\Delta \Delta \alpha_{a}(\lambda ,t)=\Delta \alpha_{a}(\lambda ,t)-\alpha_{a,\mathrm{Ret}\_500}(\lambda ,t)\;$ (Figure 5) show the absorption coefficient development due to Ret_460 isomer formation, Ret_405 formation by Ret_500 deprotonation, and Ret_340 formation due to Ret_460 deprotonation. In the short-wavelength region, λ < 300 nm, apoprotein absorption increase is observed (Figure 3, Figure 4 and Figure 5).

The isomerization and deprotonation dynamics was analyzed by a differential equation system which was solved numerically. Various absorption cross-sections, isomerization time constants, and deprotonation time constants were determined by simulating the temporal dependence of α_a_(*t*) at 580 nm, Δα_a_(*t*) at 500 nm, 460 nm, 405 nm, and 340 nm, and of ΔΔα_a_(*t*) at 460 nm, 405 nm, and 340 nm.

## 2. Materials and Methods

The QuasAr1 gene was a gift from Adam E. Cohen (Addgene plasmid # 64135, [4]). The sample preparation of QuasAr1 was reported in ref. [17]. The expressed QuasAr1 protein in the final buffer, containing 50 mM Tris-HCl (pH 8.0), 150 mM NaCl, 0.02% DDM, 0.004% CHS, 0.1 mM PMSF, and 5% glycerol, was aliquoted to amounts of 30 μL in Eppendorf tubes, shock-frozen, and stored at −80 °C until thawing for experimental investigations.

The transmission measurements, $T(\lambda )=\exp\left(-\alpha (\lambda )l\right)$, were carried out with a spectrophotometer (Cary 50, Varian Australia Pty Ltd., Mulgrave, Victoria, Australia) as described in ref. [17].

The thawed QuasAr1 solution was filled in a fused silica ultra-micro cell (inner cell size 1.5 × 3 × 5 mm^3^, from Hellma Analytics, Müllheim, Germany). The cell length for the transmission measurements was l = 3 mm. It was centrifuged with 4400 rpm for 30 min at 4 °C (Centrifuge 5702 R, Eppendorf AG, Hamburg, Germany). Then the sample was stored at room temperature (21–25 °C) in the dark and transmission spectra were measured at certain time intervals. After the absorption measurement at storage time *t* = 1200 h, the sample was centrifuged with 4400 rpm for 30 min at 4 °C, and then at *t* = 1201 h the absorption spectrum was measured again. After that, a fluorescence spectroscopic analysis occurred (see ref. [17]). Then, the sample was stored for further 51 days in the dark at room temperature before a further absorption measurement was carried out at *t* = 2424 h. Then the sample was centrifuged with 4400 rpm for 20 min at 4 °C. Finally, at *t* = 2425 h the last absorption spectrum measurement was carried out.

## 3. Results

### 3.1. Temporal Development of Attenuation Coefficient Spectra

The temporal development of attenuation coefficient spectra α(λ) of QuasAr1 in pH 8 Tris buffer at room temperature (ϑ = 21–25 °C) in the dark is presented in Figure S6 of ref. [17]. The spectra are redrawn in Figure 1 using a logarithmic attenuation coefficient ordinate (linear ordinate scale drawing is presented in Figure S1 of the Supplementary Materials). The long-wavelength part (λ > 710 nm) shows a significant rise of light attenuation between 10 h and 24 h, a leveling-off between 24 h and 456 h, and then some decrease in light attenuation. This attenuation behavior in the transparency region of QuasAr1 is due to Rayleigh scattering and Mie scattering caused by protein aggregation. With storage time, the attenuation decreased around 580 nm (PRSB, Ret_580), new attenuation built-up and decreased around 500 nm (PRSB, Ret_500) and 460 nm (PRSB, Ret_460), and it built-up around 405 nm (RSB, Ret_405) and 340 nm (RSB, Ret_340). Below 300 nm (apoprotein region) the attenuation rise is thought to be due to Mie scattering, formed retinal component absorption, and apoprotein absorption increase.

### 3.2. Mie Scattering Due to QuasAr1 Aggregation

The temporal attenuation coefficient development at λ = 750 nm in the spectral transparency region of QuasAr1 is displayed in Figure 2. Within the first 6 h no attenuation was observed. In the time range between 10 h and 24 h the light attenuation rose steeply to α = 0.105 ± 0.015 cm^−1^, then it kept approximately constant within the next 15 days, and eventually decreased to α = 0.05 ± 0.015 cm^−1^ at a storage time of *t* = 101 d.

The light attenuation coefficient α(λ) in the transparency region is equal to the light scattering coefficient α_s_(λ). The light scattering is thought to be due to QuasAr1 aggregation. For small aggregate size, diameter *d* < 0.05 λ/*n*_w_ where *n*_w_ is the refractive index of the solvent (here water), the Rayleigh scattering theory is responsible [18,19,20,21]. For larger aggregate size the Mie scattering theory is appropriate [18,19,21,22,23,24,25].

From the scattering coefficient dependence displayed in Figure 2, the QuasAr1 aggregation behavior is analyzed in Section S2 of the Supplementary Materials. There, the monomeric Rayleigh scattering cross-section, σ_R,m_(750 nm) ≈ 5 × 10^−22^ cm^2^, the aggregation scattering enhancement factor development, *M*_sca_(*t*), the degree of aggregation development, β_m_(*t*), and the refractive index of QuasAr1, *n*_Q_(750 nm) ≈ 1.6029, are determined.

### 3.3. Absorption Coefficient Development

The temporal absorption coefficient development α_a_(λ, *t*) was determined from the measured attenuation coefficient development α(λ, *t*) presented in Figure 1 by subtracting the scattering contribution α_s_(λ, *t*) according to $\alpha_{a}(\lambda ,t)=\alpha (\lambda ,t)-\alpha_{s}(\lambda ,t)$. The scattering contribution was calculated by the empirical relation $\alpha_{s}(\lambda ,t)=\alpha_{s}(\lambda_{0},t)\times {\left(\lambda_{0}/\lambda \right)}^{\gamma (t)}$ with a reference wavelength λ_0_ in the transparency region and the Mie scattering power factor γ < 4 [26,27]. The reference wavelength was set to λ_0_ = 900 nm. The Mie scattering power factor γ was adjusted to the strength of light scattering by trial and error. The applied scattering coefficient spectra, α_s_(λ, *t*), are shown in Figure S8, and the thereby used power factor dependence, γ(*t*), is shown in Figure S9 of the Supplementary Materials. The accuracy of α_s_(λ) determination reduced with decreasing wavelength because of inverse potential wavelength dependence and direct scattering observation only in the transparency region.

The obtained absorption coefficient spectra α_a_(λ, *t*) are displayed in Figure 3 with logarithmic ordinate scale (linear representation in Figure S2 of the Supplementary Materials). The initial absorption coefficient spectrum, α_a_(λ, *t* = 0) was composed of the absorption coefficient spectrum of the initially present protonated retinal Schiff base Ret_580, some residual retinals, and the apoprotein [17], i.e., $\alpha_{a}(\lambda ,t=0)=\alpha_{a,\mathrm{Ret}\_580}(\lambda ,t=0)+\alpha_{a,\mathrm{residual}\;\mathrm{retinals}}(\lambda ,t=0)+\alpha_{\mathrm{apoprotein}}(\lambda ,t=0)$. As already discussed for the attenuation spectra in Figure 1, with storage time *t* Ret_580 thermally isomerized to Ret_500 and Ret_460, and these protonated retinal Schiff bases deprotonated to Ret_405 and Ret_340 neutral retinal Schiff bases.

The absorption coefficient development at 580 nm, 500 nm, 460 nm, 405 nm, and 340 nm is displayed in Figure S3 of the Supplementary Materials. At 580 nm, which is the wavelength of peak Ret_580 absorption, the absorption initially decreased fast and then decreased slowly. Ret_580 indicates a two-component decay: one component, Ret_580_I_, isomerized fast to Ret_500, showing a fast absorption increase at 500 nm, the other component, Ret_580_II_, isomerized slowly to Ret_460. The absorption development at 500 nm, 460 nm, 405 nm, and 340 nm was caused by the isomerization of Ret_580_I_ to Ret_500 and of Ret_580_II_ to Ret_460 followed by deprotonation of Ret_500 to Ret_405 and of Ret_460 to Ret_340 (see below).

In order to better see the isomerization of Ret_580 to Ret_500 and Ret_460 as well as the deprotonation of Ret_500 to Ret_405 and of Ret_460 to Ret_340, absorption coefficient difference spectra, $\Delta \alpha_{a}(\lambda ,t)=\alpha_{a}(\lambda ,t)-\alpha_{a,\mathrm{Ret}\_580}(\lambda ,t)-\alpha_{a,\mathrm{residual}\;\mathrm{retinals}}(\lambda ,t=0)-\alpha_{a,\mathrm{apoprotein}}(\lambda ,t=0)$, are displayed in Figure 4 (linear ordinate scale presentation in Figure S4 of the Supplementary Materials). In the storage time range *t* < 117 h the difference absorption spectra development is dominated by Ret_500 formation with absorption peak at λ ≈ 500 nm. In the storage time range up to 456 h, Ret_500 formation levels off and Ret_460 formation at 460 nm gains importance. For longer storage times the absorption around 500 nm and 460 nm decreased and the absorption around 405 nm (Ret_405) and 340 nm (Ret_340) increased due to deprotonation of Ret_500 to Ret_405 and of Ret_460 to Ret_340. In Figure 4 the curve labeled ‘95 h–24 h’ belonging to $\Delta \alpha_{a}(\lambda ,95\;h)-\Delta \alpha_{a}(\lambda ,24\;h)$ resembles the shape of the absorption coefficient spectrum of Ret_500 for λ > 300 nm, because in this time range only the formation of Ret_500 is significant.

The absorption coefficient development of Ret_460, Ret_405 and Ret_340 is displayed in Figure 5 (linear ordinate scale presentation in Figure S5 of the Supplementary Materials) where absorption coefficient double difference spectra, $\Delta \Delta \alpha_{a}(\lambda ,t)=\Delta \alpha_{a}(\lambda ,t)-\alpha_{a,\mathrm{Ret}\_500}(\lambda ,t)$ are shown for different storage times listed in the legend. The absorption coefficient growth around 460 nm (Ret_460 absorption) is moderate, since Ret_580 (Ret_580_II_) isomerized slowly to Ret_460 and during its formation it deprotonated to Ret_340 showing up in the absorption around 340 nm. The absorption peaking around 405 nm was due to deprotonation of Ret_500 to Ret_405.

**Figure 6:**
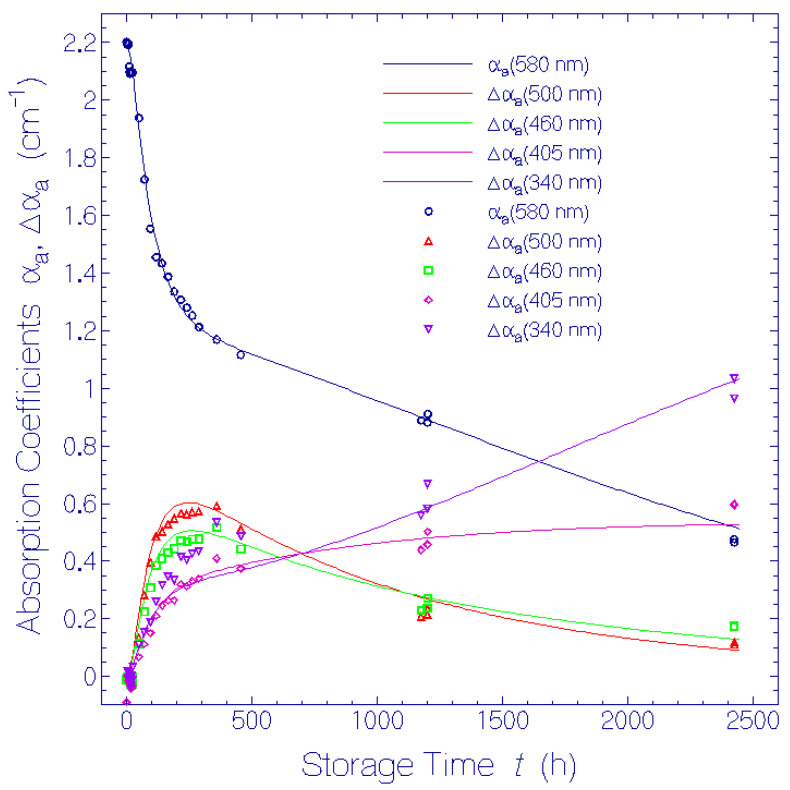
Temporal development of absorption coefficient α_a_(580 nm) and of absorption coefficient differences Δα_a_ at specific wavelengths 500 nm, 460 nm, 405 nm and 340 nm for QuasAr1 in pH 8 Tris buffer during storage in the dark at room temperature. Points are experimental results. Curves are simulations.

In Figure 6 the temporal development of the absorption coefficient, $\alpha_{a}(580\mathrm{nm},t)$, and the absorption coefficient differences, $\Delta \alpha_{a}(500\;\mathrm{nm},t)$, $\Delta \alpha_{a}(460\;\mathrm{nm},t)$, $\Delta \alpha_{a}(405\;\mathrm{nm},t)$, and $\Delta \alpha_{a}(340\;\mathrm{nm},t)$, are displayed. The symbols are experimental results from Figure 3 and Figure 4, and the curves are theoretical simulations (see below).

The absorption coefficient development α_a_(580 nm, *t*) indicates the fast ismerization of Ret_580_I_ to Ret_500 and the slow isomerization of Ret_580_II_ to Ret_460. The initial rise of the absorption coefficient difference Δα_a_(500 nm, *t*) is due to Ret_500 formation because of isomerization of Ret_580_I_, and the following decrease is due to Ret_500 deprotonation to Ret_405. The temporal dependence of Δα_a_(460 nm, *t*) is determined by the absorption behavior of Ret_500 and the absorption contribution of Ret_460 which is formed by isomerization of Ret_580_II_ to Ret_460 and depleated by subsequent deprotonation of Ret_460 to Ret_340. Δα_a_(405 nm, *t*) is determined by absorption contributions of Ret_500, Ret_460, and the Ret_405 formation by deprotonation of Ret_500. Δα_a_(340 nm, *t*) is determined by absorption contributions of Ret_500, Ret_460, Ret_405, and the Ret_340 formation by deprotonation of Ret_460.

**Figure 7:**
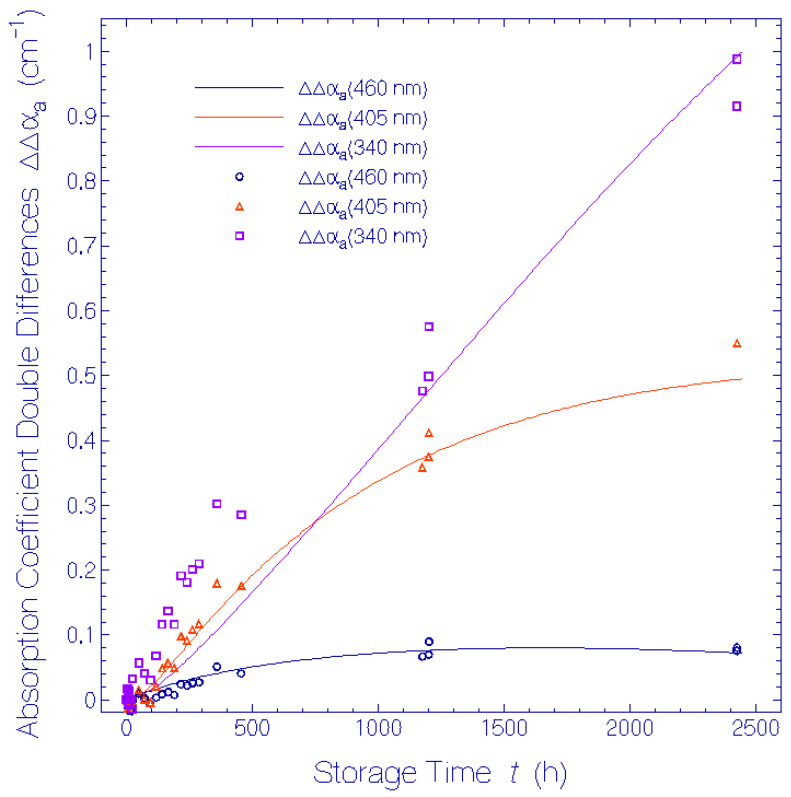
Temporal development of absorption coefficient double differences, ΔΔα_a_ at specific wavelengths 460 nm, 405 nm, and 340 nm for QuasAr1 in pH 8 Tris buffer during storage in the dark at room temperature. Points are experimental results. Curves are simulations.

In Figure 7 the temporal development of the absorption coefficient double differences, $\Delta \Delta \alpha_{a}(460\;\mathrm{nm},t)$, $\Delta \Delta \alpha_{a}(405\;\mathrm{nm},t)$, and $\Delta \Delta \alpha_{a}(340\;\mathrm{nm},t)$, are displayed. The symbols are experimental results from Figure 5 and the curves are theoretical simulations (see below).

The absorption coefficient double difference dependence at 460 nm is equal to the absorption coefficient dependence of Ret_460, i.e., $\Delta \Delta \alpha_{a}(460\;\mathrm{nm},t)=\alpha_{a,\mathrm{Ret}\_460}(460\;\mathrm{nm},t)$. The absorption coefficient double difference ΔΔα_a_(405 nm, *t*) is given by $\Delta \Delta \alpha_{a}(405\;\mathrm{nm},t)=\alpha_{a,\mathrm{Ret}\_460}(405\;\mathrm{nm},t)+\alpha_{a,\mathrm{Ret}\_405}(405\;\mathrm{nm},t)$. The absorption coefficient double difference ΔΔα_a_(340 nm, *t*) is given by the sum of the absorption coefficients of Ret_460, $\alpha_{a,\mathrm{Ret}\_460}(340\;\mathrm{nm},t)$, Ret_405, $\alpha_{a,\mathrm{Ret}\_405}(340\;\mathrm{nm},t)$, Ret_340, $\alpha_{a,\mathrm{Ret}\_340}(340\;\mathrm{nm},t)$, and other decay products, $\alpha_{a,\mathrm{other}\;\mathrm{decay}\;\mathrm{products}}(340\;\mathrm{nm},t)$.

## 4. Theoretical Thermal Absorption Dynamics Simulation of QuasAr1

### 4.1. Scheme of QuasAr1 Isomerization and Deprotonation

**Figure 8:**
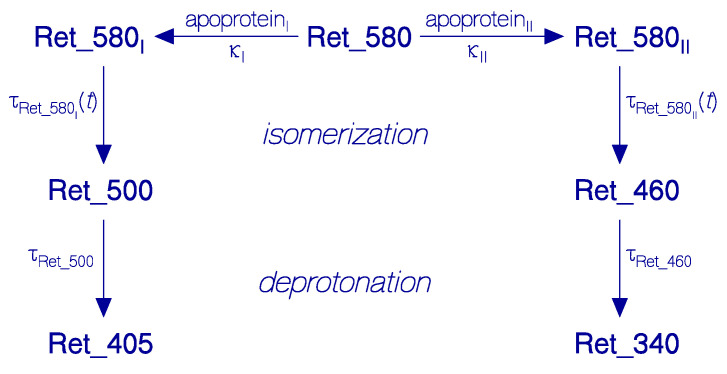
Scheme of dominant temporal dynamics of degradation of QuasAr1 in pH 8 Tris buffer in the dark at room temperature.

The dominant temporal absorption development of QuasAr1 in pH 8 Tris buffer at room temperature in the dark is modeled by the scheme of Figure 8. The originally present protonated retinal Schiff base (PRSB) Ret_580 (likely *all-trans* conformation) is composed of two differently ground-state isomerizing fractions Ret_580_I_ and Ret_580_II_ probably due to different adjacent amino acid arrangement of the apoprotein around the retinal Schiff base cofactor. Ret_580_I_ isomerizes moderately fast to Ret_500 (likely 13-*cis* conformation in apoprotein_I_ environment), and Ret_580_II_ isomerizes slowly to Ret_460 (likely 13-*cis* conformation in apoprotein_II_ environment). Ret_500 deprotonates to the neutral retinal Schiff base (RSB) Ret_405, while Ret_460 deprotonates to RSB Ret_340.

### 4.2. Numerical Simulation of QuasAr1 Thermal Dynamics

The experimental development of the protonated retinal Schiff base isomerization and subsquent deprotonation at room temperature in the dark is simulated numerically with a Fortran program using the following differential equation system for the number densities *N*_i_ of the populations in the retinal Schiff base constitutions of Figure 8.

The differential equation system for the temporal development of the level population number densities of the retinal components of Figure 8 reads (parameters are described in the symbols table at the end of the paper):

(1) $$\frac{dN_{{\mathrm{Ret}\_580}_{I}}}{dt}=-\frac{N_{{\mathrm{Ret}\_580}_{I}}}{\tau_{{\mathrm{Ret}\_580}_{I}}(t)},$$

(2) $$\frac{dN_{{\mathrm{Ret}\_580}_{\mathrm{II}}}}{dt}=-\frac{N_{{\mathrm{Ret}\_580}_{\mathrm{II}}}}{\tau_{{\mathrm{Ret}\_580}_{\mathrm{II}}}(t)},$$

(3) $$\frac{dN_{\mathrm{Ret}\_500}}{dt}=\frac{N_{{\mathrm{Ret}\_580}_{I}}}{\tau_{{\mathrm{Ret}\_580}_{I}}(t)}-\frac{N_{\mathrm{Ret}\_500}}{\tau_{\mathrm{Ret}\_500}},$$

(4) $$\frac{dN_{\mathrm{Ret}\_460}}{dt}=\frac{N_{{\mathrm{Ret}\_580}_{\mathrm{II}}}}{\tau_{{\mathrm{Ret}\_580}_{\mathrm{II}}}(t)}-\frac{N_{\mathrm{Ret}\_460}}{\tau_{\mathrm{Ret}\_460}},$$

(5) $$\frac{dN_{\mathrm{Ret}\_405}}{dt}=\frac{N_{\mathrm{Ret}\_500}}{\tau_{\mathrm{Ret}\_500}},$$

(6) $$\frac{dN_{\mathrm{Ret}\_340}}{dt}=\frac{N_{\mathrm{Ret}\_460}}{\tau_{\mathrm{Ret}\_460}},$$

with approximate isomerization time constants

(7) $$\tau_{{\mathrm{Ret}\_580}_{I}}\left(t\right)=\left(\tau_{{\mathrm{Ret}\_580}_{I}}^{0}-\tau_{{\mathrm{Ret}\_580}_{I}}^{\infty }\right)\exp\left(-\frac{t}{\delta \tau_{{\mathrm{Ret}\_580}_{I}}}\right)+\tau_{{\mathrm{Ret}\_580}_{I}}^{\infty },$$

(8) $$\tau_{{\mathrm{Ret}\_580}_{\mathrm{II}}}\left(t\right)=\left(\tau_{{\mathrm{Ret}\_580}_{\mathrm{II}}}^{0}-\tau_{{\mathrm{Ret}\_580}_{\mathrm{II}}}^{\infty }\right)\exp\left(-\frac{t}{\delta \tau_{{\mathrm{Ret}\_580}_{\mathrm{II}}}}\right)+\tau_{{\mathrm{Ret}\_580}_{\mathrm{II}}}^{\infty },$$

The intial conditions are:

(9) $$N_{\mathrm{Ret}\_580}(t=0)=N_{\mathrm{Ret}\_580,0}=\alpha_{a}(\lambda =580\;\mathrm{nm},t=0)/\sigma_{a,\mathrm{Ret}\_580}(\lambda =580\;\mathrm{nm}),$$

(10) $$N_{{\mathrm{Ret}\_580}_{I}}(t=0)=\kappa_{I}N_{\mathrm{Ret}\_580}(t=0),$$

(11) $$N_{{\mathrm{Ret}\_580}_{\mathrm{II}}}(t=0)=\kappa_{\mathrm{II}}N_{\mathrm{Ret}\_580}(t=0),$$

(12) $$N_{\mathrm{Ret}\_500}(t=0)=0,$$

(13) $$N_{\mathrm{Ret}\_460}(t=0)=0,$$

(14) $$N_{\mathrm{Ret}\_405}(t=0)=0,$$

(15) $$N_{\mathrm{Ret}\_340}(t=0)=0.$$

$N_{\mathrm{Ret}\_580}(t=0)=N_{\mathrm{Ret}\_580,0}$ = 1.381 × 10^−16^ cm^−3^ is determined using absorption coefficient $\alpha_{a}(\lambda =580\;\mathrm{nm},t=0)$ = 2.20 cm^−1^ (Figure 3) and absorption cross-section $\sigma_{a,\mathrm{Ret}\_580}(\lambda =580\;\mathrm{nm})$ = 1.593 × 10^−16^ cm^2^ (Figure S3 of [17] and Figure S10 of Supplementary Materials).

The unknown parameters are: κ_I_, the initial fraction of Ret_580_I_ in Ret_580; κ_II_, the initial fraction of Ret_580_II_ in Ret_580; $\tau_{{\mathrm{Ret}\_580}_{I}}^{0}$, the initial time constant of Ret_580_I_ isomerization; $\tau_{{\mathrm{Ret}\_580}_{I}}^{\infty }$, the final time constant of Ret_580_I_ isomerization; $\delta \tau_{{\mathrm{Ret}\_580}_{I}}$, the exponential time constant of change-over from initial to final Ret_580_I_ isomerization; $\tau_{{\mathrm{Ret}\_580}_{\mathrm{II}}}^{0}$, the initial time constant of Ret_580_II_ isomerization; $\tau_{{\mathrm{Ret}\_580}_{\mathrm{II}}}^{\infty }$, the final time constant of Ret_580_II_ isomerization; $\delta \tau_{{\mathrm{Ret}\_580}_{\mathrm{II}}}$, the exponential time constant of change-over from initial to final Ret_580_II_ isomerization; τ_Ret_500_, the time constant of Ret_500 deprotonation; and τ_Ret_460_, the time constant of Ret_460 deprotonation.

The unknown parameters are determined by numerical fitting the experimental storage time development of the absorption coefficients $\alpha_{a}(\lambda =580\;\mathrm{nm},t)$, the absorption coefficient differences $\Delta \alpha_{a}(\lambda =500\;\mathrm{nm},t)$, $\Delta \alpha_{a}(\lambda =460\;\mathrm{nm},t)$, $\Delta \alpha_{a}(\lambda =405\;\mathrm{nm},t)$, $\Delta \alpha_{a}(\lambda =340\;\mathrm{nm},t)$, and the absorption coefficient double differences $\Delta \Delta \alpha_{a}(\lambda =460\;\mathrm{nm},t)$, $\Delta \Delta \alpha_{a}(\lambda =405\;\mathrm{nm},t)$, and $\Delta \Delta \alpha_{a}(\lambda =340\;\mathrm{nm},t)$.

The absorption coefficent developments are given by

(16) $$\alpha_{a}(\lambda =580\;\mathrm{nm},t)=\left(N_{{\mathrm{Ret}\_580}_{I}}(t)+N_{{\mathrm{Ret}\_580}_{\mathrm{II}}}(t)\right)\sigma_{a,\mathrm{Ret}\_580}(\lambda =580\;\mathrm{nm}),$$

(17) $$\Delta \alpha_{a}(\lambda =500\;\mathrm{nm},t)=N_{\mathrm{Ret}\_500}(t)\sigma_{a,\mathrm{Ret}\_500}(\lambda =500\;\mathrm{nm})+N_{\mathrm{Ret}\_460}(t)\sigma_{a,\mathrm{Ret}\_460}(\lambda =500\;\mathrm{nm}),$$

(18) $$\begin{array}{ll} \Delta \alpha_{a}(\lambda =460\;\mathrm{nm},t)= & N_{\mathrm{Ret}\_500}(t)\sigma_{a,\mathrm{Ret}\_500}(\lambda =460\;\mathrm{nm})+N_{\mathrm{Ret}\_460}(t)\sigma_{a,\mathrm{Ret}\_460}(\lambda =460\;\mathrm{nm})\\ & +N_{\mathrm{Ret}\_405}(t)\sigma_{a,\mathrm{Ret}\_405}(\lambda =460\;\mathrm{nm}) \end{array},$$

(19) $$\begin{array}{ll} \Delta \alpha_{a}(\lambda =405\;\mathrm{nm},t)= & N_{\mathrm{Ret}\_500}(t)\sigma_{a,\mathrm{Ret}\_500}(\lambda =405\;\mathrm{nm})+N_{\mathrm{Ret}\_460}(t)\sigma_{a,\mathrm{Ret}\_460}(\lambda =405\;\mathrm{nm})\\ & +N_{\mathrm{Ret}\_405}(t)\sigma_{a,\mathrm{Ret}\_405}(\lambda =405\;\mathrm{nm}) \end{array},$$

(20) $$\begin{array}{ll} \Delta \alpha_{a}(\lambda =340\;\mathrm{nm},t)= & N_{\mathrm{Ret}\_500}(t)\sigma_{a,\mathrm{Ret}\_500}(\lambda =340\;\mathrm{nm})+N_{\mathrm{Ret}\_460}(t)\sigma_{a,\mathrm{Ret}\_460}(\lambda =340\;\mathrm{nm})\\ & +N_{\mathrm{Ret}\_405}(t)\sigma_{a,\mathrm{Ret}\_405}(\lambda =340\;\mathrm{nm})+N_{\mathrm{Ret}\_340}(t)\sigma_{a,\mathrm{Ret}\_340}(\lambda =340\;\mathrm{nm}) \end{array},$$

(21) $$\begin{array}{ll} \Delta \Delta \alpha_{a}(\lambda =460\;\mathrm{nm},t)= & N_{\mathrm{Ret}\_460}(t)\sigma_{a,\mathrm{Ret}\_460}(\lambda =460\;\mathrm{nm})\\ & +N_{\mathrm{Ret}\_405}(t)\sigma_{a,\mathrm{Ret}\_405}(\lambda =460\;\mathrm{nm}) \end{array},$$

(22) $$\begin{array}{ll} \Delta \Delta \alpha_{a}(\lambda =405\;\mathrm{nm},t)= & N_{\mathrm{Ret}\_460}(t)\sigma_{a,\mathrm{Ret}\_460}(\lambda =405\;\mathrm{nm})\\ & +N_{\mathrm{Ret}\_405}(t)\sigma_{a,\mathrm{Ret}\_405}(\lambda =405\;\mathrm{nm}) \end{array},$$

(23) $$\begin{array}{l} \Delta \Delta \alpha_{a}(\lambda =340\;\mathrm{nm},t)=N_{\mathrm{Ret}\_460}(t)\sigma_{a,\mathrm{Ret}\_460}(\lambda =340\;\mathrm{nm})\\ \;\;\;\;+N_{\mathrm{Ret}\_405}(t)\sigma_{a,\mathrm{Ret}\_405}(\lambda =340\;\mathrm{nm})\;+N_{\mathrm{Ret}\_340}(t)\sigma_{a,\mathrm{Ret}\_340}(\lambda =340\;\mathrm{nm}) \end{array}$$

The simulated curves, α_a_(λ = 580 nm, *t*), Δα_a_(λ = 500 nm, *t*), Δα_a_(λ = 460 nm, *t*), Δα_a_(λ = 405 nm, *t*), and Δα_a_(λ = 340 nm, *t*) are shown in Figure 6, and the simulated curves, ΔΔα_a_(λ = 460 nm, *t*), ΔΔα_a_(λ = 405 nm, *t*), and ΔΔα_a_(λ = 340 nm, *t*) are shown in Figure 7. The applied parameters in the simulations are collected in Table 1. The ±-digits indicate uncertainties of the values.

**Figure 9:**
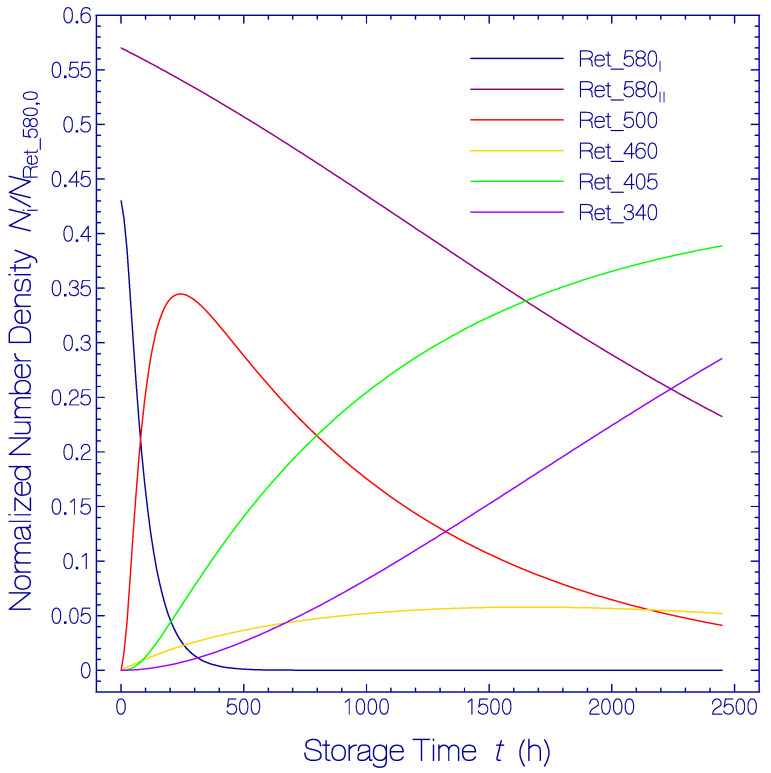
Temporal normalized population number density development, *N*_i_(*t*)*N*_Ret_580,0,_ of QuasAr1 constituents *i* = Ret_580_I_, Ret_580_II,_ Ret_500, Ret_460, Ret_405, and Ret_340. *N*_Ret_580,0_ = *N*_Ret_580_(*t =* 0).

The calculated normalized population number density development *N*_i_/*N*_Ret_580,0_ versus storage time *t* is displayed in Figure 9. Thereby *i* stands for Ret_580_I_, Ret_580_II_, Ret_500, Ret_460, Ret_405, and Ret_340. *N*_Ret_580,0_ stands for the initial Ret_580 number density at *t* = 0. The number density of Ret_580_I_ decreases fast and causes the fast rise in the number density of Ret_500. Ret_500 decays slowly to Ret_405. Ret_580_II_ decreases slowly and causes the formation of Ret_460. The number density of Ret_460 remains small since Ret_460 decays with faster time constant to Ret_340 than it is formed. Ret 405 and Ret_340 continously grow up.

## 5. Discussion

The room temperature dynamics of the Archaerhodopsin 3 based fluorescent voltage sensor QuasAr1 studied in [17] has been reinvestigated, applying improved light scattering contibution subtraction and numerical simulation of the absorption development. The previously proposed dynamics of Ret_580_II_ isomerization to Ret_640 (absorption peak at 640 nm) with fast Ret_640 deprotonation to Ret_350 could not be simulated. Refined scattering contribution subtraction led to the disappearance of the absorption coefficient difference Δα_a_(*t*) around 640 nm shown in Figure 7b of ref. [17] (see new Figure S4 in the Supplementary Materials).

The attenuation coefficient development at λ = 750 nm depicted in Figure 2 indicates the onset of light scattering after a storage time of 6 h approaching a scattering plateau within about 24 h. This attenuation coefficient development is interpreted in Section S2 of the Supplementary Materials as QuasAr1 aggregation, and Rayleigh scattering and Mie scattering theory was applied there to gain information on the aggregation dynamics. The degree of aggegation approached β_m_ ≥ 14,000, and aggregate particle radius *a*_ag_ ≥ 46 nm. The QuasAr1 protein refractive index was calculated to be *n*_Q_ = 1.6029.

The absorption coefficient spectra development, α_a_(λ, *t*), is shown in Figure 3, where the scattering contibution to the attenuation coeffcient spectra was subtracted, applying an empirical scattering coefficient spectra formula (Section S2.3 and Equation (S12) of the Supplementary Materials). To visualize the Ret_580 isomerization dynamics, the absorption coefficient difference spectra, Δα_a_(λ, *t*), were calculated subtracting the Ret_580 contributions (Figure 4). The buildup of the isomer Ret_500 by Ret_580_I_ isomerization within the first 300 h and its later decay due to deprotonation of Ret_500 to Ret_405 is clearly seen. In Figure 5 the absorption coefficient double difference spectra, ΔΔα_a_(λ, *t*), are shown, obtained by subtracting the Ret_500 absorption coeffcient contributions. They reveal the formation of a weak absorption band around λ = 460 nm due to isomerization of Ret_580_II_ to Ret_460. Around λ = 405 nm the buildup of Ret_405 as a deprotonation product of Ret_500 can be seen clearly. The absorption band around λ = 340 nm is due to fast deprotonation of Ret_460 to Ret_340. The absorption rise below λ = 300 nm is dominantly attributed to inceasing apoprotein absorption due to apoprotein modification with storage time.

In Figure 6 the α_a_(580 nm, *t*) dependence clearly shows the two-component degradation of Ret_580 (fast isomerization of Ret_580_I_ to Ret_500, and slow isomerization of Ret_580_II_ to Ret_460). The Δα_a_(500 nm, *t*) and Δα_a_(460 nm, *t*) dependences show the Ret_500 and Ret_460 buildups and decays. The Δα_a_(405 nm, *t*) and Δα_a_(340 nm, *t*) curves indicate the formation of the neutral retinal Schiff bases Ret_405 and Ret_340 due to deprotonation of Ret_500 and Ret_460, respectively.

In Figure 7 the ΔΔα_a_(460 nm, *t*) curve is equal to α_a,Ret_460_(460 nm, *t*) and shows the Ret_460 development with storage time *t*. The ΔΔα_a_(405 nm, *t*) curve was determined by the absorption of Ret_405 with a small contribution from Ret_460. The ΔΔα_a_(340 nm, *t*) curve was dominated by the absorption of Ret_340 with contributions from Ret_405 and Ret_460.

The isomerization dynamics of Ret_580_I_ and Ret_580_II_ and the deprotonation dynamics of the isomer products Ret_500 and Ret_460 were described by a differential equation system which was solved numerically. The involved absorption cross-sections and the time parameters were determined by fitting the calulations to the experimental results. Absorption cross-section spectra of Ret_580, Ret_500, Ret_460 and Ret_405 are shown in Figure S10 of Section S3 of the Supplementary Materials. The Ret_580 decay time development is presented in Figure S11 of Section S4 of the Supplementary Materials. It is thought that it was caused by structural changes of the QuasAr1 apoprotein with storage time.

The fit of calculated curves to the experimental data in Figure 6 and Figure 7 is reasonably good. Only the numerical curve ΔΔα_a_(λ = 340 nm, *t*) does not fit well to the experimental points, indicating additional minor isomerization and deprotonation paths of the originally present (Ret_580_I_, Ret_580_II_) and formed (Ret_500; Ret:460) constituents of QuasAr1 which are not included the model.

## 6. Conclusions

The previously invested absorption spectroscopic thermal dynamics of the Archaerhodopsin 3 based fluorescent voltage sensor QuasAr1 [17] was reanalyzed in detail and was numerically simulated with a differental equation system. The combined experimemtal and theoretical investigation allows model developments and avoids misinterpretations. This makes it possible to calculate quantitative reaction paramters.

The absorption spectroscopic studies performed at room temperature in the dark enable us to evaluate the thermal stability of the sample under investigation; this is a prerequiste to carrying out purpose applications of interest, such as the investigation of the photocycle dynamics of QuasAr1 [28]. The presented findings indicate that QuasAr1 can be used for approximately 10 h in its initial form before the onset of modifications due to aggregation and ground-state isomerization.

The thermal dynamics results obtained here for QuasAr1 cannot be used to estimate the thermal behavior of other variants of Archaerhodopsin 3 based voltage sensors, as is shown by the results obtained for Archon2 [29] (there was no indication of protonated retinal Schiff base ground-state isomerization and there was an onset of light scattering after two days due to beginning of protein unfolding). The thermal stability of each microbial rhodopsin based voltage sensor needs to be investigated individually.

## Figures and Tables

**Table 1:** Parameters for QuasAr1 in pH 8 Tris buffer at room temperature in the dark.

Parameter	Value	Comment
κ_I_	0.43 ± 0.02	In Equation (10)
κ_II_	0.57 ± 0.02	In Equation (11)
$\tau_{{\mathrm{Ret}\_580}_{I}}^{0}$	450 ± 50 h	In Equation (7)
$\tau_{{\mathrm{Ret}\_580}_{I}}^{\infty }$	80 ± 10 h	In Equation (7)
$\delta \tau_{{\mathrm{Ret}\_580}_{I}}$	13 ± 1 h	In Equation (7)
$\tau_{{\mathrm{Ret}\_580}_{\mathrm{II}}}^{0}$	5000 ± 300 h	In Equation (8)
$\tau_{{\mathrm{Ret}\_580}_{\mathrm{II}}}^{\infty }$	1700 ± 100 h	In Equation (8)
$\delta \tau_{{\mathrm{Ret}\_580}_{\mathrm{II}}}$	1000 ± 50 h	In Equation (8)
τ_Ret_500_	1000 ± 50 h	In Equation (3)
τ_Ret_460_	400 ± 30 h	In Equation (4)
σ_Ret_580_(580 nm)	(1.593 ± 0.05) × 10^−16^ cm^2^	See Figure S10, and [17]
σ_Ret_500_(500 nm)	(1.25 ± 0.05) × 10^−16^ cm^2^	See Figure S10
σ_Ret_500_(460 nm)	(1.0 ± 0.05) × 10^−16^ cm^2^	See Figure S10
σ_Ret_500_(405 nm)	(5.47 ± 0.05) × 10^−17^ cm^2^	See Figure S10
σ_Ret_500_(340 nm)	(5.85 ± 0.1) × 10^−17^ cm^2^	See Figure S10
σ_Ret_460_(500 nm)	(2.64 ± 0.05) × 10^−17^ cm^2^	See Figure S10
σ_Ret_460_(460 nm)	(1.0 ± 0.05) × 10^−16^ cm^2^	See Figure S10
σ_Ret_460_(405 nm)	(5.34 ± 0.1) × 10^−17^ cm^2^	See Figure S10
σ_Ret_460_(340 nm)	(4.40 ± 0.2) × 10^−17^ cm^2^	See Figure S10
σ_Ret_405_(460 nm)	(1.60 ± 0.1) × 10^−18^ cm^2^	See Figure S10
σ_Ret_405_(405 nm)	(8.5 ± 0.5) × 10^−17^ cm^2^	See Figure S10
σ_Ret_405_(340 nm)	(3.72 ± 0.5) × 10^−17^ cm^2^	See Figure S10
σ_Ret_340_(340 nm)	(1.8 ± 0.3) × 10^−16^ cm^2^	In Equation (23)
*N*_Ret_580,0_	(1.381 ± 0.05) × 10^16^ cm^−3^	In Equation (9)

## Data Availability

The original contributions presented in this study are included in the article/Supplementary Material. Further inqueries can be directed to the corresponding author.

## Supplementary Materials

The following supporting information can be downloaded at: https://www.mdpi.com/article/10.3390/bioengineering12121293/s1, Figure S1: Temporal development of attenuation coefficient spectra of QuasAr1 in pH 8 Tris buffer at room temperature in the dark; Figure S2: Temporal development of absorption coefficient spectra of QuasAr1 in pH 8 Tris buffer at room temperature in the dark; Figure S3: Temporal development of absorption coefficients at wavelengths 580 nm, 500 nm, 460 nm, 405 nm, and 340 nm of QuasAr1 in pH 8 Tris buffer at room temperature in the dark; Figure S4: Temporal development of absorption coefficient difference spectra of QuasAr1 in pH 8 Tris buffer at room temperature in the dark; Figure S5: Temporal development of absorption coefficient double difference spectra of QuasAr1 in pH 8 Tris buffer at room temperature in the dark; Figure S6: Scattering cross-section at 750 nm versus storage time for QuasAr1 in pH 8 Tris buffer at room temperature in the dark; Figure S7: Temporal development of aggregation scattering enhancement factor; Figure S8: Calculated scattering coefficient spectra of QuasAr1 in pH 8 Tris buffer at room temperature in the dark for various storage times; Figure S9: Variation of adjusted Mie scattering power factor with storage time for sample of QuasAr1 in pH 8 Tris buffer at room temperature in the dark; Figure S10: Absorption cross-section spectra of Ret_580, Ret_500, Ret_460 and Ret_405; Figure S11: Temporal development of time constants of isomerization of Ret_580_I_ and Ret_580_II_; Table S1: Scattering relevant parameters of Quasar1 at 750 nm; Table S2: Amino acid numbers, molar refractivity values, volume densities, and molar masses of constituents of QuasAr1. References [30,31,32,33,34,35,36,37,38] are cited in the Supplementary Materials.

## Abbreviations

Arch	Archaerhodopsin 3 from *Halorubrum sodmense*	
GEVI	Genetically encoded voltage indicator	
PRSB	Protonated retinal Schiff base	
QuasAr	Quality superior to Arch	
Ret_xxx	Retinal with absorption maximum approximately at xxx nm	
RSB	Retinal Schiff base	
**Symbols**		
**Parameter**	**Unit**	**Meaning**
a_ag_	nm	Aggregate radius
a_m_	nm	Monomer radius
$\tilde{M}$		Total Mie scattering function
*M*_m_	g mol^−1^	Molar mass
*M*_sca_		Aggregation scattering enhancement factor
*N*_0_	cm^−3^	QuasAr1 number density
*N*_Ret_580,0_	cm^−3^	Initial number density of Ret_580 at *t* = 0
*N*_Ret_580,I_	cm^−3^	Number density of Ret_580_I_
*N*_Ret_580,II_	cm^−3^	Number density of Ret_580_II_
*N*_Ret_xxx_	cm^−3^	Number density of Ret_xxx
*n*_w_		Refractive index of water (solvent)
*n*_Q_		Refractive index of QuasAr1 (solute)
*t*	h	Storage time
α	cm^−1^	Attenuation coefficient
α_a_	cm^−1^	Absorption coefficient
α_s_	cm^−1^	Scattering coefficient
Δα_a_	cm^−1^	Absorption coefficient difference
ΔΔα_a_	cm^−1^	Absorption coefficient double difference
β_m_		Degree of aggregation
γ		Mie scattering power factor
κ_I_		Initial fraction of Ret_580_I_ in Ret_580
κ_II_		Initial fraction of Ret_580_II_ in Ret_580
λ	nm	Vacuum wavelength
σ_a_	cm^2^	Absorption cross-section
σ_R,ag_	cm^2^	Rayleigh aggregate scattering cross-section
σ_R,m_	cm^2^	Rayleigh monomer scattering cross-section
σ_s_	cm^2^	Scattering cross-section
$\tau_{{\mathrm{Ret}\_580}_{I}}$	h	Isomerization time constant of Ret_580_I_
$\tau_{{\mathrm{Ret}\_580}_{\mathrm{II}}}$	h	Isomerization time constant of Ret_580_II_
τ_Ret_500_	h	Deprotonation time constant of Ret_500
τ_Ret_460_	h	Deprotonation time constant of Ret_460
