# Supplementary material for: Reinvestigation of Absorption Spectroscopic Thermal Dynamics of Archaerhodopsin 3 Based Voltage Sensor QuasAr1
Source: Bioengineering (Basel). 2025 Nov 24;12(12):1293. doi: 10.3390/bioengineering12121293 (PMC12729394; doi:10.3390/bioengineering12121293)
Supplement: Supplementary file 1 [file bioengineering-12-01293-s001.zip › Bioengineering-3972398-SM.pdf]

bioengineering

MDPI

Article

Supplementary Materials: Reinvestigation of Absorption Spectroscopic Thermal Dynamics of Archaerhodopsin 3 Based Voltage Sensor QuasAr1

Alfons Penzkofer [1*], Arita Silapetere [2] and Peter Hegemann [2]

[1] Fakultät für Physik, Universität Regensburg, Universitätsstraße 31, D-93053 Regensburg, Germany

[2] Experimentelle Biophysik, Institut für Biologie, Humboldt Universität zu Berlin, Invalidenstraße 42, D-10115 Berlin, Germany

S1. Attenuation coefficient spectra and derived absorption coefficient spectra

The attenuation coefficient spectra of QuasAr1 in linear ordinate scale are shown in Figure S1 (taken from [17]). The attenuation in the long-wavelength transparency region (λ > 700 nm) indicates occurrence of light scattering.

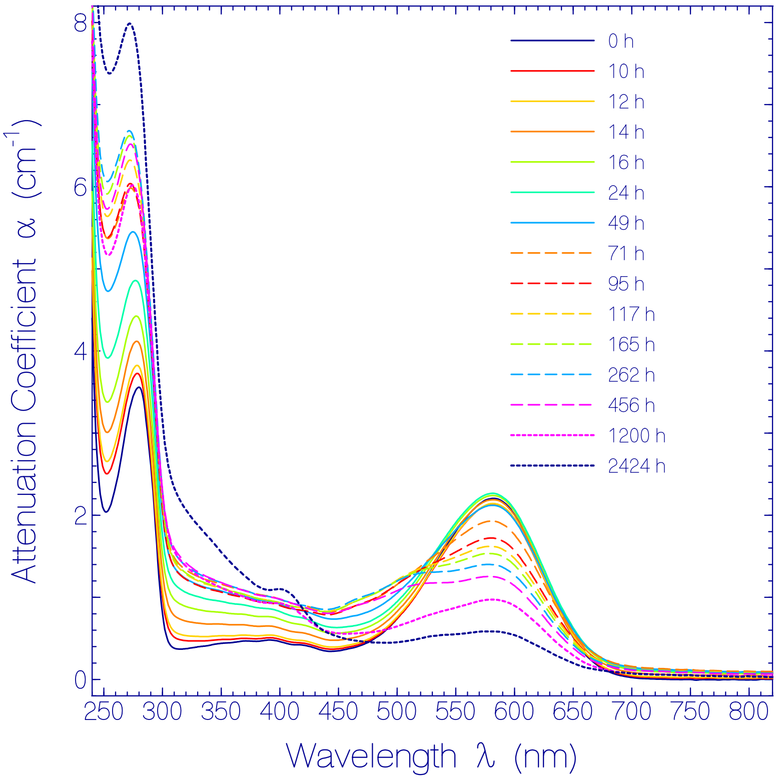

Figure S1. Temporal development of attenuation coefficient spectra α(λ) of QuasAr1 in pH 8 Tris buffer at room temperature in the dark (from [17]). The storage times are listed in the legend.

The absorption coefficient spectra (Figure 3) in linear ordinate scale are presented in Figure S2. They are determined by careful Mie scattering contribution subtraction. The absorption coefficient spectra of Ret_580, residual retinals, and apoprotein of QuasAr1 at storage time t = 0 are included.

 https://doi.org/10.3390/bioengineering12121293

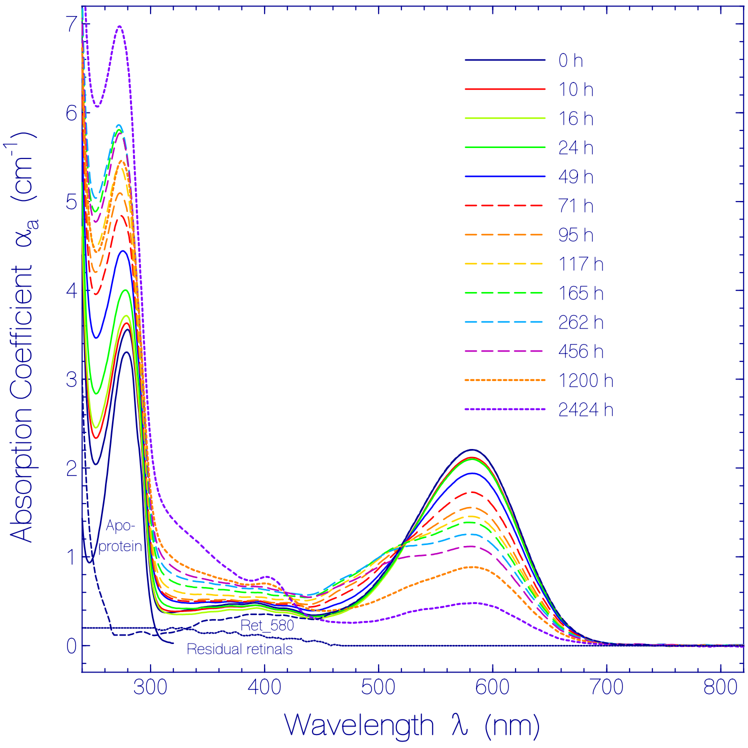

Figure S2. Temporal development of absorption coefficient spectra $\alpha_a(\lambda)$ of QuasAr1 in pH 8 Tris buffer at room temperature in the dark. The storage times are listed in the legend.

The temporal absorption coefficient development at λ = 580 nm (absorption maximum $\alpha_{a,max}$ of Ret_580), 500 nm ($\alpha_{a,max}$ of Ret_500), 460 nm ($\alpha_{a,max}$ of Ret_460), 405 nm ($\alpha_{a,max}$ of Ret_405), and 340 nm ($\alpha_{a,max}$ of Ret_340) are displayed in Figure S3.

After the absorption measurement at t = 1200 h, the sample was centrifuged with 4400 rpm for 30 min at 4 °C, and then at t = 1201 h the absorption spectrum was measured again. After that, a fluorescence spectroscopic analysis occurred. Following, the sample was stored for a further 51 days in the dark at room temperature before a further absorption measurement was carried out at t = 2424 h. Then, the sample was centrifuged with 4400 rpm for 20 min at 4 °C. Finally, at t = 2425 h the last absorption spectrum measurement was carried out.

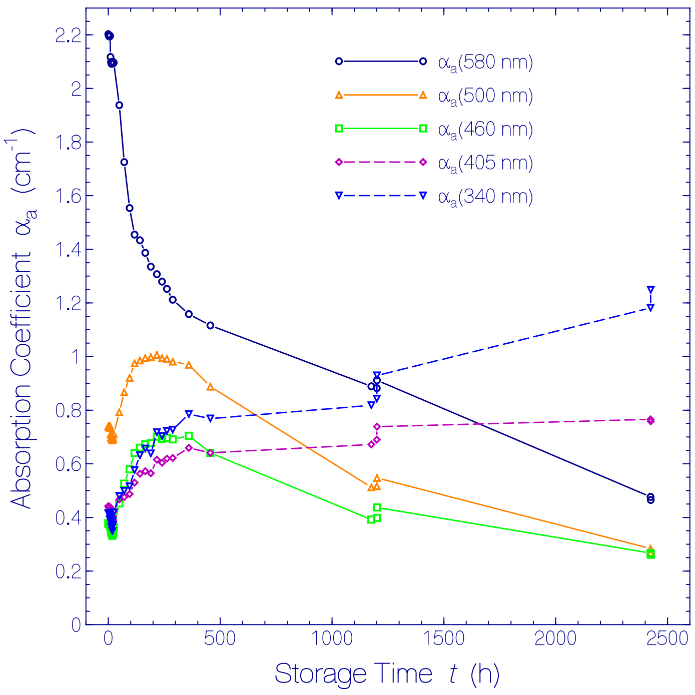

Figure S3. Temporal development of absorption coefficients α_a(580 nm), α_a(500 nm), α_a(460 nm), α_a(405 nm), and α_a(340 nm) of QuasAr1 in pH 8 Tris buffer at room temperature in the dark.

The absorption coefficient difference spectra $\Delta\alpha_a(\lambda, t)$ (Figure 4) in linear ordinate scale are presented in Figure S4. The Ret_580, residual retinal, and initial apoprotein absorption coefficient contributions, $\alpha_{a,\mathrm{Ret\_580}}(\lambda, t)$, $\alpha_{a,\mathrm{residual\ retinals}}(\lambda, t = 0)$, and $\alpha_{a,\mathrm{apoprotein}}(\lambda, t = 0)$, were subtracted.

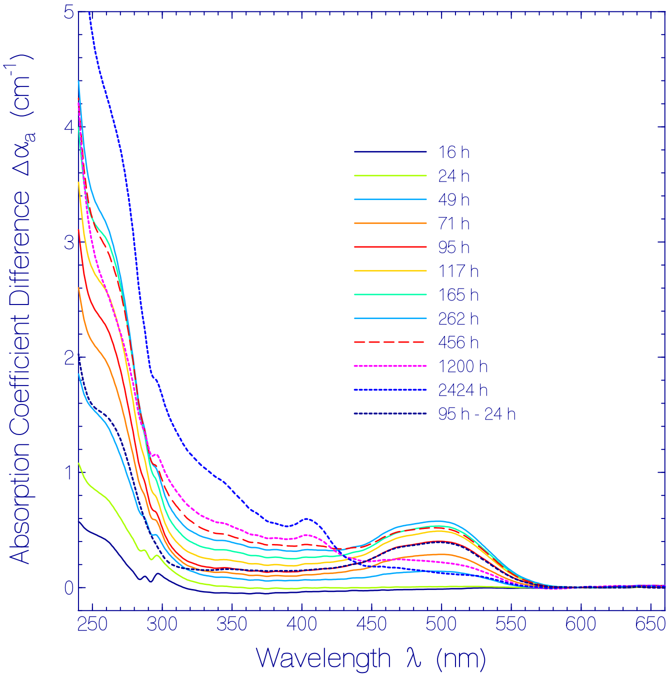

Figure S4. Temporal development of absorption coefficient difference spectra $\Delta\alpha_a(\lambda, t)$ of QuasAr1 in pH 8 Tris buffer at room temperature in the dark. The storage times are listed in the legend.

The absorption coefficient double difference spectra $\Delta\Delta\alpha_a(\lambda, t)$ (Figure 5) in linear ordinate scale are presented in Figure S5. The Ret_500 absorption coefficient contributions, $\alpha_{a,\mathrm{Ret\_500}}(\lambda, t)$, were subtracted.

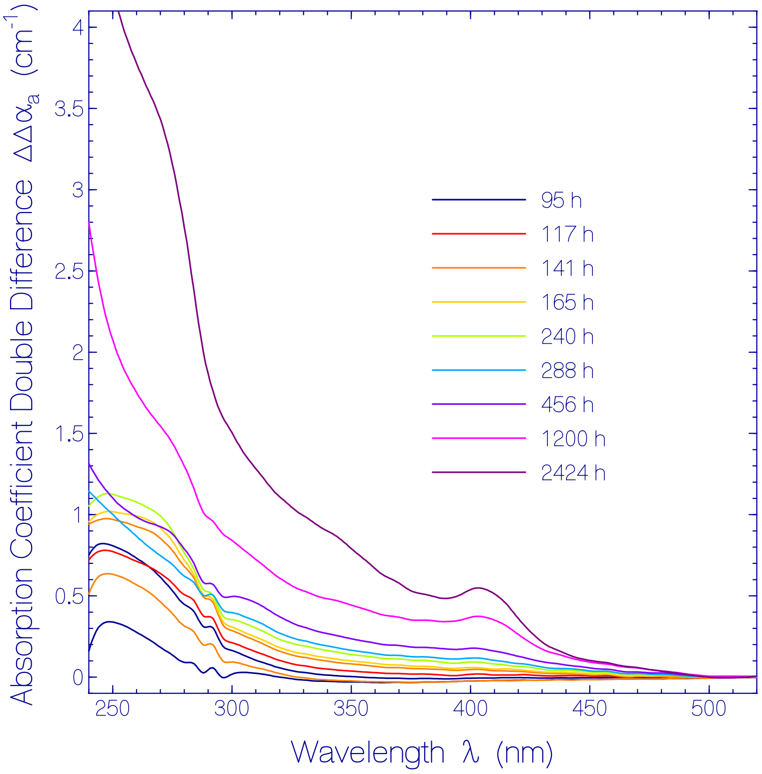

Figure S5. Temporal development of absorption coefficient double difference spectra $\Delta\Delta\alpha(\lambda)$ of QuasAr1 in pH 8 Tris buffer at room temperature in the dark. The storage times are listed in the legend.

S2. Scattering considerations

S2.1. Aggregation scattering enhancement factor and degree of aggregation

Light scattering manifests itself by light transmission loss in the transparency region of the investigated compound. Here, the attenuation coefficient α is equal to the scattering coefficient α_s. For QuasAr1 in pH 8 Tris buffer at room temperature in the dark, it shows up by the temporal attenuation coefficient development for $\lambda > 710$ nm in Figure 1 and Figure S1. As an example, the attenuation coefficient α (equal to the scattering coefficient α_s) development at λ = 750 nm versus storage time t is displayed in Figure 2.

The scattering coefficient α_s is related to the scattering cross-section σ_s by

$$\alpha_s(\lambda) = N_0\sigma_s(\lambda) \quad \text{(S1)}$$

where N_0 is the QuasAr1 number density. N_0 was determined in [17] from the apoprotein absorption. For the used sample in Figure 1 it was determined to be $N_0 = N_{\mathrm{Ret\_580}}(t=0) + N_{\mathrm{Residual\ retinals}}(t=0)$ = 1.60×10^{16} cm^{-3}. In Figure S6 the scattering coefficient dependence of Figure 2, α(750 nm, t), was redrawn to the absorption cross-section dependence σ_s(750 nm, t) for storage time t > 6h by use of Equation (S1). For $t \leq 6$ h the calculated QuasAr1 monomer Rayleigh scattering cross-section is displayed (see below). The σ_s(750 nm, t) dependence is used in the following to discuss the temporal QuasAr1 aggregation behavior by application of the forward Rayleigh and Mie scattering theory.

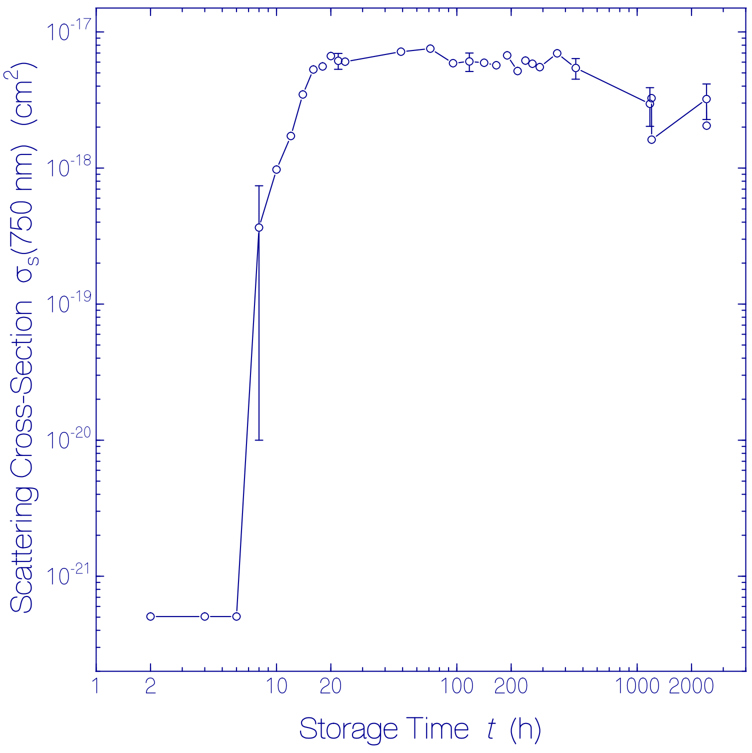

Figure S6: Scattering cross-section σ_s at λ =750 nm versus storage time *t* for QuasAr1 in pH 8 Tris buffer at room temperature in the dark.

The Rayleigh scattering and Mie scattering was described in [23,24]. The scattering cross-section is given by

$$\sigma_s = \sigma_{R,ag}\tilde{M} = \sigma_{R,m}M_{sca} = \sigma_{R,m}\beta_m\tilde{M} = \frac{8\pi}{3}\frac{d\sigma_{R,m}^{fw}}{d\Omega}\beta_m\tilde{M}\,, \tag{S2}$$

where $\sigma_{R,ag}$ is the Rayleigh aggregate scattering cross-section, $\sigma_{R,m}$ is the Rayleigh monomer scattering cross-section, $M_{sca} = \beta_m\tilde{M}$ is the aggregation scattering enhancement factor, β_m is the degree of aggregation (number of molecules forming an aggregate), $\tilde{M} \le 1$ is the total Mie scattering function, and $d\sigma_{R,m}^{fw}/d\Omega$ is the monomeric differential forward Rayleigh scattering cross-section, which is given by [23,24]

$$\frac{d\sigma_{R,m}^{fw}}{d\Omega} = \frac{4\pi^2 n_w^4}{\lambda^4}v_m^2\left|\frac{m^2-1}{m^2+2}\right|^2, \tag{S3}$$

where n_w is the refractive index of the solvent (here water) at the vacuum wavelength λ, v_m is the molecule volume, $m = \tilde{n}_Q / n_w$ is the complex refractive index ratio of solute (here QuasAr1) $\tilde{n}_Q$ to solvent n_w. The complex refractive index $\tilde{n}_Q = n_Q - i\kappa_Q$ reduces to the refractive index n_Q in the transparency region where the extinction coefficient is κ_Q = 0. The extinction coefficient κ is related to the absorption coefficient α_a by the relation $\kappa = \alpha_a / (4\pi\tilde{\nu})$ where $\tilde{\nu} = \lambda^{-1}$ is the wavenumber (p. 522 in [30], p. 88 in [31]).

The monomer molecular volume v_m is given by

$$v_m = \frac{4\pi}{3}a_m^3 = \frac{M_m}{N_A\rho_m}, \tag{S4}$$

where a_m is the spherical monomer radius, M_m is the monomer molar mass, N_A is the Avogadro constant, and ρ_m is the monomer mass density.

The relevant parameters involved in Equation (S1)–Equation (S4) for QuasAr1 are collected in Table S1. The QuasAr1 refractive index is determined below in Section S2.2. The resulting $M_{sca}(t) = \beta_m(t)\tilde{M}(t) = \sigma_s(t)/\sigma_{R,m}$ dependence is displayed in Figure S7. Since $\tilde{M}(t) \le 1$, the degree of aggregation is $\beta_m(t) \ge M_{sca}(t)$.

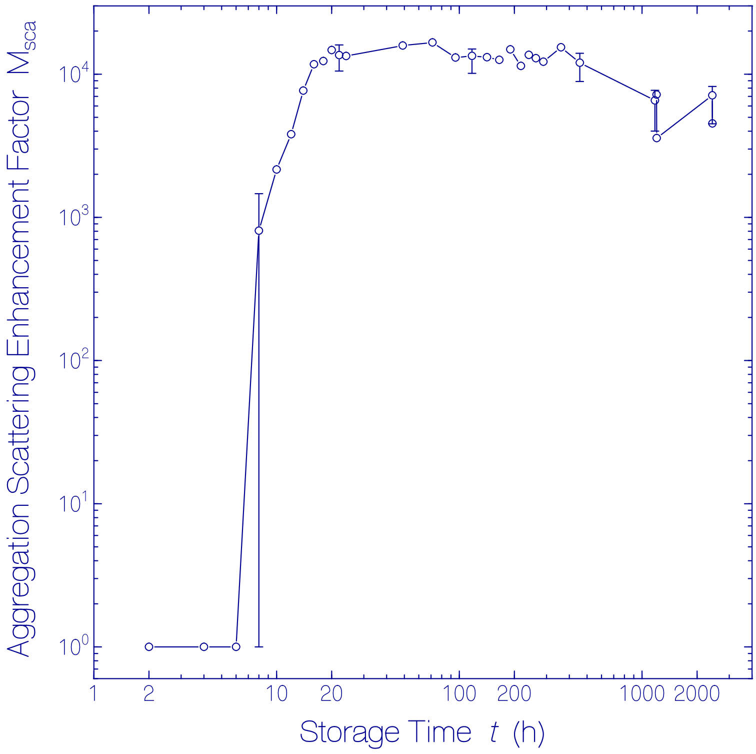

Figure S7. Development of aggregation scattering enhancement factor M_{sca} versus storage time *t* of QuasAr1 in pH 8 Tris buffer at room temperature in the dark.

As seen in Figure S7, within one day of sample storage in the dark at room temperature, the aggregation scattering enhancement factor grew up to $M_{sca} \approx 14000$, corresponding to a degree of aggregation $\beta_m \ge 14000$. The corresponding aggregate volume was $v_{ag} = \beta_m v_m \ge 4.2\times10^{-16}$ cm^3, and the corresponding aggregate radius was $a_{ag} \ge 46.5$ nm.

Table S1. Scattering relevant parameters of QuasAr1 at λ = 750 nm

**Parameter**	**Value**	**Reference**
Refractive index n_w	1.3300	[32]
Refractive index n_Q	1.6029	Section S2.2
Molar mass M_m	25831.44 g mol^{-1}	$M_m = M_{apo} + M_{PRSB}$
M_{apo}	25548 g mol^{-1}	[17]
M_{PRSB}	283.44 g mol^{-1}	[17]
Monomer mass density ρ_m	1.430 g cm^{-3}	[33]

Monomer volume v_m	3.0×10^{-20} cm^3	Equation (S4)
Monomer radius a_m	1.927 nm	Equation (S4)
Monomeric differential forward Rayleigh scattering cross-section $d\sigma_{R,m}^{fw}/d\Omega$	6.035×10^{-23} cm^{2}sr^{-1}	Equation (S3)
Monomer Rayleigh scattering cross-section $\sigma_{R,m} = (8\pi/3) d\sigma_{R,m}^{fw}/d\Omega$	5.056×10^{-22} cm^2	Equations (S2) and (S3)

S2.2. Determination of refractive index of QuasAr1

The refractive index n_Q of QuasAr1 entered the light scattering analysis. It was determined by the Clausius Mossotti equation (Lorentz-Lorenz formula) [34]

$$R_Q = \frac{N_A}{3\varepsilon_0}\alpha_Q = V_Q \frac{n_Q^2 - 1}{n_Q^2 + 2}, \tag{S5}$$

where R_Q is the molar refractivity of QuasAr1, $N_A = 6.02205\times10^{23}$ mol^{-1} is the Avogadro constant, $\varepsilon_0 = 8.854188\times10^{-12}$ CV^{-1}m^{-1} is the vacuum permittivity, α_Q is the molecular polarizability of QuasAr1, and V_Q is the molar volume of QuasAr1. Solving Equation (S5) for n_Q gives

$$n_Q = \left(\frac{V_Q + 2R_Q}{V_Q - R_Q}\right)^{1/2}. \tag{S6}$$

R_Q is composed of the molar refractivity values of the apoprotein R_{apo} and the protonated retinal Schiff base (PRSB) of QuasAr1 according to

$$R_Q = R_{apo} + R_{PRSB}. \tag{S7}$$

R_{apo} is given by the sum of molar refractivity values of the composing amino acids of QuasAr1 [35, 36]

$$R_{apo} = \sum_i \kappa_i R_i, \tag{S8}$$

where the sum runs over all present amino acids in QuasAr1. κ_i counts the number of amino acid residues *i*, and R_i is the molar refractivity of amino acid residue *i*, (the amino acid sequence of QuasAr1 is given in Figure S1 of [17]). The amino acid composition numbers κ_i and the individual molar refractivity values R_i are compiled in Table S2. The result is $R_{apo} = 7644.21$ cm^{3}mol^{-1}.

The molar refractivity of PRSB is calculated from the reported theoretical molecular polarizability of PRSB given in [37,38]. The average polarizability determined in [38] is $\alpha_{PRSB} = (\alpha_{xx} + \alpha_{yy} + \alpha_{zz})/3$ = 238.64 au = 3.93466×10^{-39} Cm^{2}V^{-1} (conversion factor: 1 au = 1.648777×10^{-41} C^{2}m^{2}J^{-1} = 1.648777×10^{-41} Cm^{2}V^{-1}, see: https://www.nist.gov/pml/fundamental-physical-constants). The corresponding molar refractivity is $R_{PRSB} = N_A\alpha_{PRSB}/(3\varepsilon_0)$ = 8.92×10^{-5} m^{3}mol^{-1} = 89.2 cm^{3}mol^{-1}.

The complete molar refractivity is $R_Q = 7733.41$ cm^{3}mol^{-1}.

The molar volume V_Q of QuasAr1 is given by the sum of the molar volumes of the constituents, apoprotein *apo* and cofactor PRSB:

$$V_Q = V_{apo} + V_{PRSB}. \tag{S9}$$

The apoprotein molar volume is given by

$$V_{apo} = \sum_i \kappa_i v_i M_i = \sum_i \kappa_i \frac{M_i}{\rho_i}, \quad \text{(S10)}$$

where v_i is the volume density of amino acid residue i (in cm^3g^{-1}), ρ_i is the mass density of amino acid residue i (in $g\ cm^{-3}$), and M_i is the molar mass of amino acid residue i (in $g\ mol^{-1}$). The v_i and M_i values for QuasAr1 are included in Table S2. The result is $V_{apo} = 22317.46\ cm^3mol^{-1}$

The molar volume of PRSB is given by

$$V_{\mathrm{PRSB}} = v_{\mathrm{PRSB}} M_{\mathrm{PRSB}} = \frac{M_{\mathrm{PRSB}}}{\rho_{PRSB}}. \quad \text{(S11)}$$

The specific volume $v_{\mathrm{PRSB}} = \rho_{\mathrm{PRSB}}^{-1}$ is assumed to be $v_{\mathrm{PRSB}} \approx 0.71\ cm^3g^{-1}$. The molar mass of PRSB is $M_{PRSB} = 283.44\ g\ mol^{-1}$. The result is $V_{PRSB} = 201.24\ cm^3mol^{-1}$.

The complete molar volume is $V_Q = 22518.70\ cm^3mol^{-1}$.

Insertion of $R_Q = 7733.41\ cm^3mol^{-1}$ and $V_Q = 22518.70\ cm^3mol^{-1}$ into Equation (S6) gives for the refractive index of QuasAr1 a value of $n_Q = 1.6029$.

Table S2. Amino acid numbers κ_i, molar refractivity values R_i (from [35,36]), volume densities v_i (from [35,36]), and molar masses M_i of constituents of QuasAr1.

Component	κ_i	R_i (cm^3mol^{-1})	v_i (cm^3g^{-1})	M_i ($g\ mol^{-1}$)
Ala (A)	27	17.15	0.74	71.09
Arg (R)	10	39.47	0.70	156.19
Asn (N)	2	26.09	0.62	114.11
Asp (D)	12	26.06	0.60	115.09
Cys (C)	1	48.58	0.63	103.15
Gln (Q)	3	30.37	0.67	128.14
Glu (E)	12	30.07	0.66	129.12
Gly (G)	27	12.81	0.64	57.05
His (H)	8	34.62	0.67	137.14
Ile (I)	19	31.87	0.90	113.16
Leu (L)	44	31.59	0.90	113.16
Lys (K)	4	34.10	0.82	128.17
Met (M)	7	34.45	0.75	131.19
Phe (F)	11	42.21	0.77	147.18
Pro (P)	7	23.74	0.76	97.12
Ser (S)	13	19.16	0.63	87.08
Thr (T)	22	23.82	0.70	87.08
Trp (W)	7	55.24	0.74	186.21
Tyr (Y)	12	44.34	0.71	163.18
Val (V)	23	26.73	0.86	99.14
PRSB	1	89.20 [38]	0.71	283.44

S2.3. Spectral scattering coefficient development

The experimental temporal light scattering coefficient development, $\alpha_s(\lambda, t)$, in the transparency region of QuasAr1 in the wavelength range $\lambda > 710$ nm can be seen in Figure 1. Over the whole spectral region of Figure 1 it is calculated by the empirical relation [26,27]

$$\alpha_s(\lambda, t) = \alpha_s(\lambda_0, t) \times \left(\frac{\lambda_0}{\lambda}\right)^{\gamma(t)} \quad \text{(S12)}$$

where λ_0 is a selected wavelength in the transparency region (here selected $\lambda_0 = 900$ nm), and $\gamma \leq 4$ is an adjusted Mie scattering power factor.

The calculated $\alpha_s(\lambda, t)$ spectra are displayed in Figure S8 and the thereby used, adjusted $\gamma(t)$ values are shown in Figure S9.

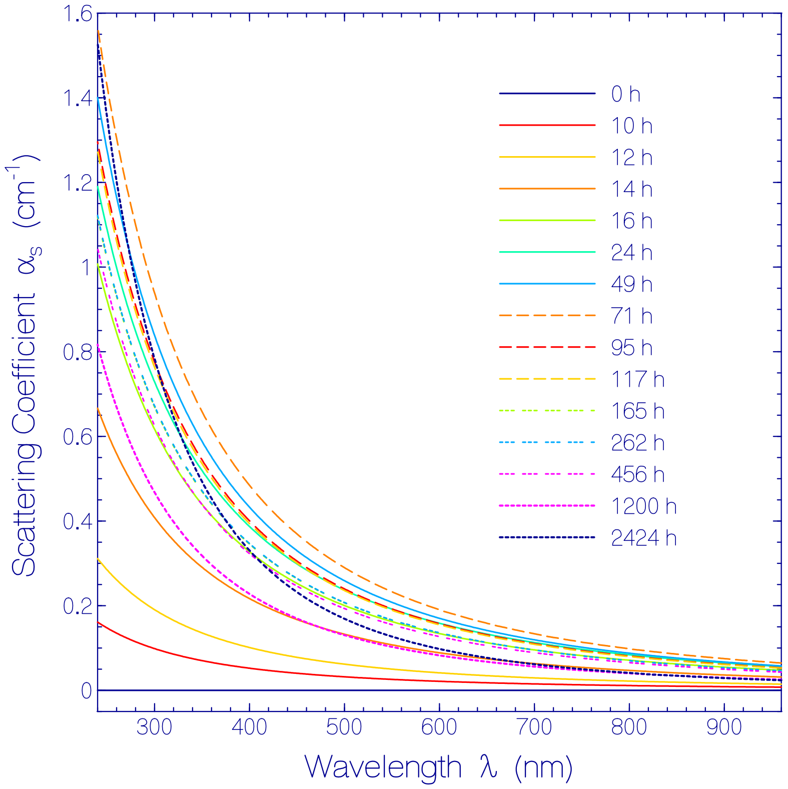

Figure S8. Calculated scattering coefficient spectra $\alpha_s(\lambda)$ of QuasAr1 in pH 8 Tris buffer at room temperature in the dark. The corresponding storage times t of the curves are listed in the legend.

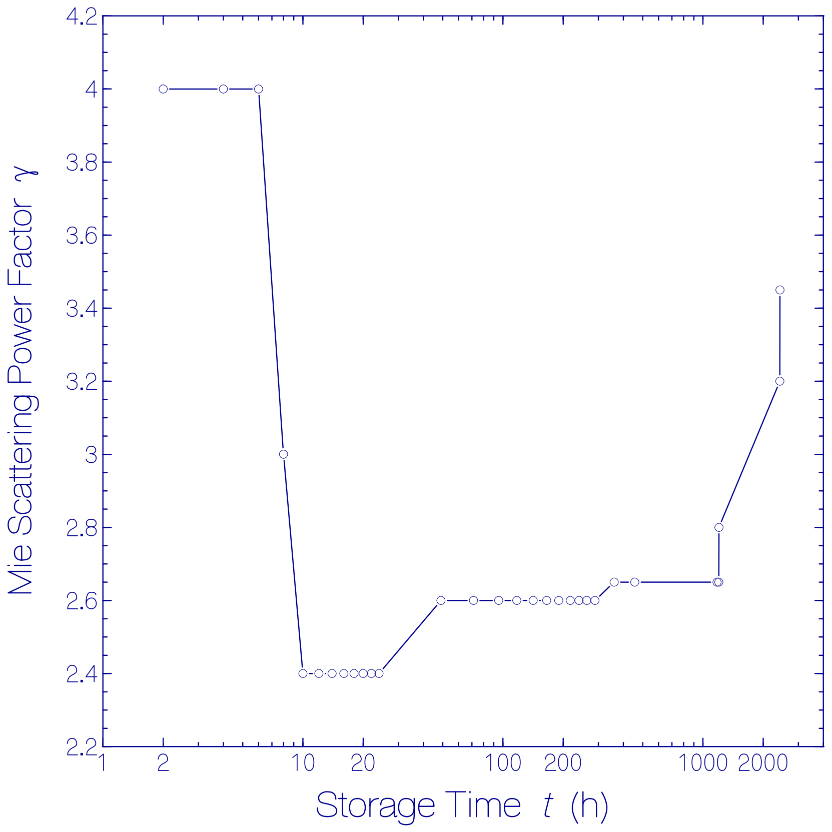

Figure S9. Variation of adjusted Mie scattering power factor γ with storage time *t* for sample of QuasAr1 in pH 8 Tris buffer at room temperature in the dark.

S3. Absorption cross-section spectra of QuasAr1 constituents

The absorption cross-section spectrum of Ret_580 was determined in ref. [17] and is shown in Figure S10. The shape of the absorption cross-section spectrum of Ret_500 was set equal to the shape of the absorption coefficient difference spectrum $\Delta\alpha_a(\lambda, t = 95\text{ h}) - \Delta\alpha_a(\lambda, t = 24\text{ h})$ of Figure 4, since in the storage time range between t = 24 h and t = 95 h practically only Ret_500 is formed, i.e., $\alpha_{a,\text{Ret\_500}}(\lambda, t = 95\text{ h}) - \alpha_{a,\text{Ret\_500}}(\lambda, t = 24\text{ h}) \approx \Delta\alpha_a(\lambda, t = 95\text{ h}) - \Delta\alpha_a(\lambda, t = 24\text{ h})$. The absolute scale was determined by the numerical simulations giving $\sigma_{a,\text{Ret\_500}}$(500 nm) = 1.25×10^{-16} cm^2. The shapes of the absorption cross-section spectra $\sigma_{a,\text{Ret\_460}}(\lambda)$ and $\sigma_{a,\text{Ret\_405}}(\lambda)$ were determined by assuming the same shape for Ret_500, Ret_460, and Ret_405 in wavenumber space and only shifting the peak wavenumber positions ($\tilde{\nu} = \lambda^{-1}$). The absolute scales are determined by the numerical simulations giving $\sigma_{a,\text{Ret\_460}}$(460 nm) = 1.0×10^{-16} cm^2 and $\sigma_{a,\text{Ret\_405}}$(405 nm) = 8.5×10^{-17} cm^2.

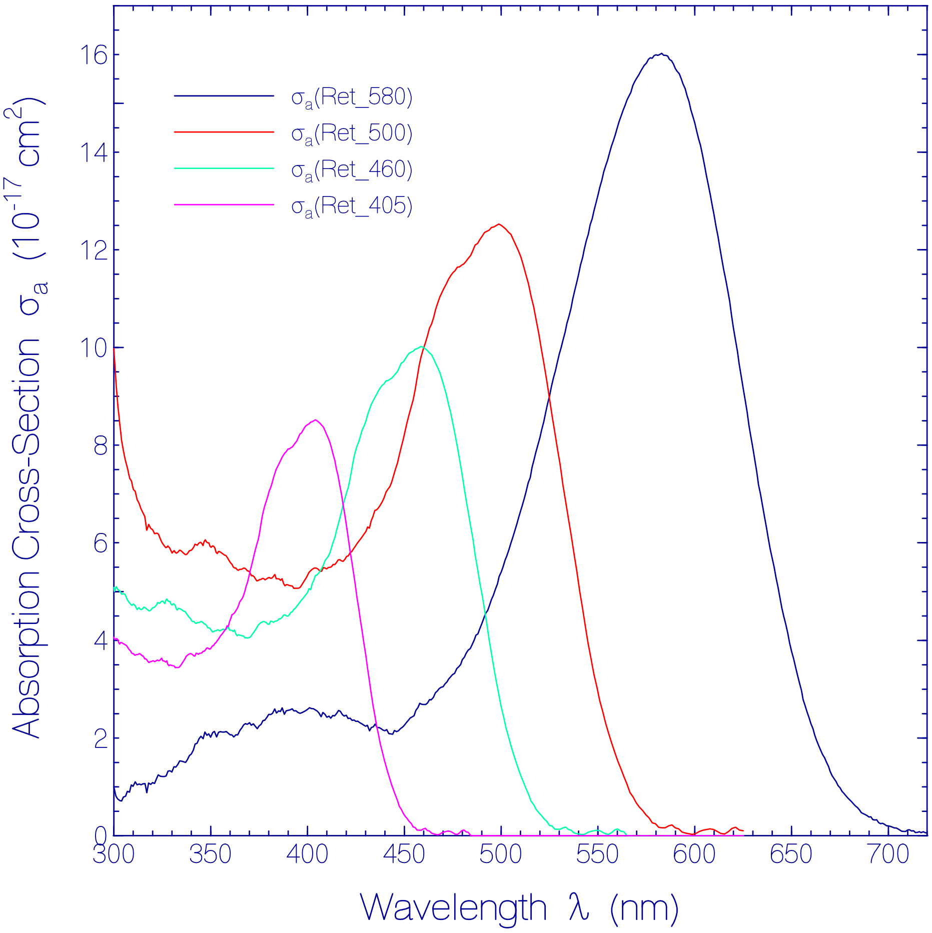

Figure S10. Absorption cross-section spectra of Ret_580, Ret_500, Ret_460 and Ret_405.

S4. Ret_580 decay time development

Ret_580 isomerizes in a two-component manner, with a storage time dependent decay time behavior, as approximated by the decay time dependencies of Equation (7) for $\tau_{\mathrm{Ret\_580_I}}$ and of Equation (8) for $\tau_{\mathrm{Ret\_580_{II}}}$. The simulated dependences are displayed in Figure S11.

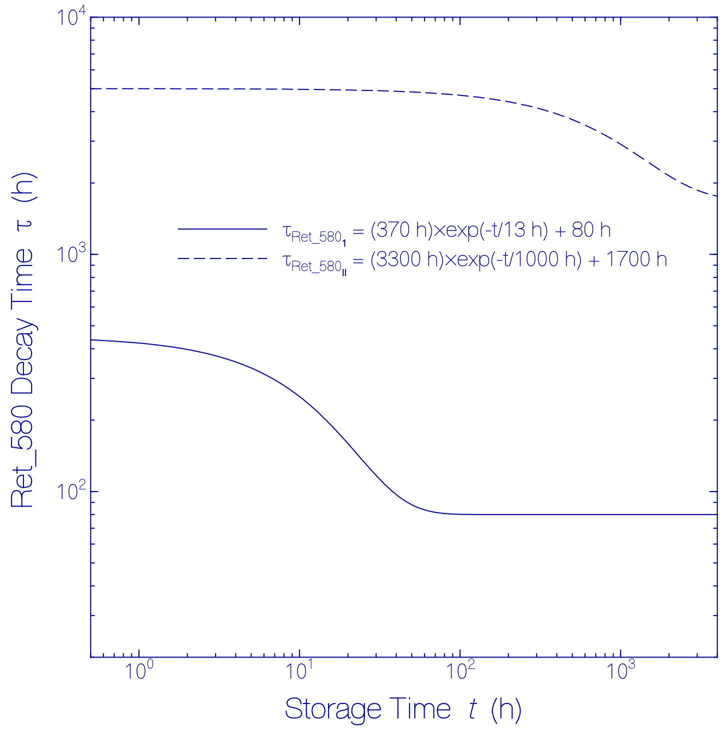

Figure S11. Temporal development of time constants of isomerization of Ret_580I, $\tau_{Ret\_580_I}$, and of Ret_580II, $\tau_{Ret\_580_{II}}$.

Disclaimer/Publisher's Note: The statements, opinions and data contained in all publications are solely those of the individual author(s) and contributor(s) and not of MDPI and/or the editor(s). MDPI and/or the editor(s) disclaim responsibility for any injury to people or property resulting from any ideas, methods, instructions or products referred to in the content.
